# An Holistic Extension for Classical Logic via Quantum Fredkin Gate

**DOI:** 10.3390/e23091178

**Published:** 2021-09-07

**Authors:** Hector Freytes, Giuseppe Sergioli

**Affiliations:** 1 Department of Mathematics, University of Cagliari, Via Ospedale 72, I-09124 Cagliari, Italy; hfreytes@gmail.com; 2 Department of Philosophy, University of Cagliari, Via Is Mirrionis 1, I-09123 Cagliari, Italy

**Keywords:** Fredkin quantum gate, bipartite quantum systems, fuzzy logic

## Abstract

An holistic extension for classical propositional logic is introduced in the framework of quantum computation with mixed states. The mentioned extension is obtained by applying the quantum Fredkin gate to non-factorizable bipartite states. In particular, an extended notion of classical contradiction is studied in this holistic framework.

## 1. Introduction

The idea of quantum computing was introduced at the beginning of the 1980s by Richard Feynman [1]. He focused on the computational benefits that arise by using quantum systems in place of classical ones. Standard quantum computation is based on quantum systems described by finite dimensional Hilbert spaces, starting from $C^{2}$, which is the two-dimensional space where the qubits reside. Indeed, a qubit is usually represented as a unit vector in $C^{2}$. As in the classical case, it is possible to introduce a certain number of *quantum logical gates* (quantum gates for short). As happens in the classical case, a quantum circuit is given by a composition of quantum gates. These gates are formally represented by unitary operators that act on qubits.

From a logical point of view, several quantum gates are interesting because they provide fuzzy and holistic extensions of the classical propositional logic. In this work, we focus our investigation on the propositional structure arising from the quantum Fredkin gate (or F for short) [2,3]. Syntactically, F can be seen as a ternary connective that is functionally complete with respect to the classical propositional logic. Moreover, the study of the logical systems based on F becomes essential since they can be directly applied to important protocols such as error correction and optimal cloning.

The aim of this paper is to introduce an holistic semantic extending the classical logic arising from F. This extension is based on the fuzzy behavior of F and on the holistic nature of bipartite quantum systems. More precisely, we first introduce a fuzzy extension of classic AND, OR and NOT connectives based on the ternary arity of F. Then, the holistic extension for the classical logic arises from the fuzzy extensions of AND, OR and NOT acting on non-separable states. In particular, in this holistic extension, the notion of contradiction is studied.

The paper has the following structure: in Section 2, an holistic-type description for bipartite quantum states is introduced. In Section 3, we provide some basic information about quantum computation. Section 4 is devoted to describing general logical systems arising from quantum gates, which is the aim of the quantum computational logic [4,5,6,7]. In Section 5, a logical system based on the quantum Fredkin gate is introduced. Moreover, a connection between the compositional logic related to the quantum Fredkin gate and the fuzzy logic of continuous *t*-norm [8,9,10] is established. In Section 6, an extension of the classical logic, arising from a fuzzification of the classical connectives {¬,∧} via the quantum Fredkin gate, is provided. In this way, the notions of classical logical consequence, contradiction and tautology are naturally extended in this framework. In Section 7, based on the fuzzy extension via the quantum Fredkin gate applied on bipartite quantum states, an holistic extension for the classical logic is established. Finally, in Section 8, we study a natural extension of the notion of classical contradiction in this holistic-type logical framework. A particular subfamily of quantum states, called three-parameter qubit states, is studied as an example of holistic contradictions.

## 2. The Holistic Component of a Bipartite Quantum System

The intuitive notion about *holism* is based on the idea that the whole is different with respect to the sum of the parts. In this section, we introduce the standard mathematical framework of the holistic feature related to bipartite quantum systems, which is helpful to describe an holistic extension of classical logic via Fredkin quantum gate.

We consider the Pauli matrices

$$\sigma_{1}=\left[\begin{array}{cc} 0 & 1\\ 1 & 0 \end{array}\right],\;\sigma_{2}=\left[\begin{array}{cc} 0 & -i\\ i & 0 \end{array}\right],\;\sigma_{3}=\left[\begin{array}{cc} 1 & 0\\ 0 & -1 \end{array}\right]\;I=\left[\begin{array}{cc} 1 & 0\\ 0 & 1 \end{array}\right]$$

where *I* is the identity matrix in $C^{2}$. It is well known that each density operator ρ over $C^{2}$ can be represented as

$$\rho =\frac{1}{2}\left(I+s_{1}\sigma_{1}+s_{2}\sigma_{2}+s_{3}\sigma_{3}\right)$$

where $s_{1},s_{2}$ and $s_{3}$ are three real numbers such that $s_{1}^{2}+s_{2}^{2}+s_{3}^{2}\leq 1$. A very similar representation can be obtained for density operators over $C^{n}$ by considering the generalized Pauli matrices introduced in the following definition.

**Definition** **1.** *Let H be an n-dimensional Hilbert space and let us consider the standard orthonormal basis of H given by $\{|\psi_{1}\rangle ,\ldots ,|\psi_{n}\rangle \}$. Let k and j be two natural numbers such that: 1≤k<j≤n. Then, it is possible to define the generalized Pauli matrices as follows:*

$${}^{\left(n\right)}\sigma_{1}^{\left[k,j\right]}=|\psi_{j}\rangle \langle \psi_{k}\left|+\right|\psi_{k}\rangle \langle \psi_{j}|$$

$${}^{\left(n\right)}\sigma_{2}^{\left[k,j\right]}=i(|\psi_{j}\rangle \langle \psi_{k}\left|-\right|\psi_{k}\rangle \langle \psi_{j}|)$$

*and for 1≤k≤n−1*

$${}^{\left(n\right)}\sigma_{3}^{\left[k\right]}=\sqrt{\frac{2}{k(k+1)}}(|\psi_{1}\rangle \langle \psi_{1}\left|+\ldots +\right|\psi_{k}\rangle \langle \psi_{k}\left|-k\right|\psi_{k+1}\rangle \langle \psi_{k+1}|).$$

If $H=C^{2}$ one immediately obtains: ${}^{\left(2\right)}\sigma_{1}^{\left[1,2\right]}=\sigma_{1}$, ${}^{\left(2\right)}\sigma_{2}^{\left[1,2\right]}=\sigma_{2}$ and ${}^{\left(2\right)}\sigma_{3}^{\left[1\right]}=\sigma_{3}.$

Let us consider ρ as a density operator belonging to the *n*-dimensional Hilbert space H. For any *j*, where $1\leq j\leq n^{2}-1$, let

$$s_{j}\left(\rho \right)=tr\left(\rho \sigma_{j}\right).$$

Then, by considering the Schlienz–Mahler representation [11], ρ can be written as:

(1) $$\rho =\frac{1}{n}I^{\left(n\right)}+\frac{1}{2}\sum_{j=1}^{n^{2}-1}s_{j}\left(\rho \right)\sigma_{j}$$

where $I^{\left(n\right)}$ is the well-known n×n identity matrix. The Schlienz–Mahler representation allows us to express an arbitrary quantum bipartite state in terms of a sum of a factorizable state with the addition of a parameter that represents, let us say, an holistic component of the state.

Now, we consider the Hilbert space $H=H_{a}⊗H_{b}$. For an arbitrary density operator ρ∈H, let us denote with $\rho_{a}$ the partial trace of ρ with respect to the sub-system $H_{b}$ (i.e., $\rho_{a}=tr_{H_{b}}\left(\rho \right)$) and with $\rho_{b}$ the partial trace of ρ with respect to the sub-system $H_{a}$ (i.e., $\rho_{b}=tr_{H_{a}}\left(\rho \right)$). The partial traces $\rho_{a}$ and $\rho_{b}$ have a matrix representation described in the following:

Let us assume that dim(H)=n, $dim(H_{a})=m$ and $dim(H_{b})=k$. It is possible to write the matrix ρ in terms of m×m block matrices $B_{i,j}$; each of them is a *k*-square matrix. In this way, we have:

(2) $$\begin{array}{ccc} \rho_{a} & = & tr_{H_{b}}\left(\rho \right)=\left[\begin{array}{cccc} trB_{1,1} & trB_{1,2} & \ldots & trB_{1,m}\\ trB_{2,1} & trB_{2,2} & \ldots & trB_{2,m}\\ \vdots & \vdots & \vdots & \vdots \\ trB_{m,1} & trB_{m,2} & \ldots & trB_{m,m} \end{array}\right] \end{array}$$

(3) $$\begin{array}{ccc} \rho_{b} & = & tr_{H_{a}}\left(\rho \right)=\sum_{i=1}^{m}B_{i,i}. \end{array}$$

**Definition** **2.** *Let us consider ρ as an arbitrary density operator in $H_{m}⊗H_{k}$ and let $dim(H_{m})=m$ and $dim(H_{k})=k$. Then, we say that ρ is (m,k)-factorizable if $\rho =\rho_{m}⊗\rho_{k}$, where $\rho_{m}$ is a density operator in $H_{m}$ and $\rho_{k}$ is a density operator in $H_{k}$, respectively.*

Let us remark that if ρ is (m,k)-factorizable as $\rho =\rho_{m}⊗\rho_{k}$, then this factorization is unique and $\rho_{m}$ and $\rho_{k}$ are the reduced states of ρ with respect to $H_{m}$ and $H_{k}$ [12].

Let $H=H_{a}⊗H_{b}$, with $dim(H_{a})=m$, $dim(H_{b})=k$. Let ρ be the density operator on H and let us consider the generalized Pauli matrices $\sigma_{1}^{a},\ldots ,\sigma_{m^{2}-1}^{a}$ and $\sigma_{1}^{b},\ldots ,\sigma_{k^{2}-1}^{b}$ coming from $H_{a}$ and $H_{b}$, respectively. Now, we introduce the coefficients

(4) $$M_{j,l}\left(\rho \right)=tr\left(\rho \left[\sigma_{j}^{a}⊗\sigma_{l}^{b}\right]\right)-tr\left(\rho \left[\sigma_{j}^{a}⊗I^{\left(k\right)}\right]\right)tr\left(\rho \left[I^{\left(m\right)}⊗\sigma_{l}^{b}\right]\right).$$

If we take into account the matrix

(5) $$M\left(\rho \right)=\frac{1}{4}\sum_{j=1}^{m^{2}-1}\sum_{l=1}^{k^{2}-1}M_{j,l}\left(\rho \right)\left(\sigma_{j}^{a}⊗\sigma_{l}^{b}\right)$$

then it is possible to prove that

(6) $$\rho =\rho_{a}⊗\rho_{b}+M\left(\rho \right).$$

In this way, Equation (6) provides a technical description of an instance of the holism related to bipartite quantum systems. Indeed, the state ρ in $H=H_{a}⊗H_{b}$ does not depend uniquely on the reduced states $\rho_{a}$ and $\rho_{b}$, but also depends on the factor M(ρ). Thus, M(ρ) represents an “additional component” of ρ, in the case where ρ is a non-factorized state. Let us notice that the matrix M(ρ) has not the form of a density operator and then it is not the representation of any physical state. We say that M(ρ) is the *holisitic component* of ρ.

## 3. Mathematical Models of Quantum Computing

In this section, we provide a brief description of the mathematical model of quantum computation and quantum gates needed for this work. Quantum computation is an extension of the classical one, where new primitive information resources are introduced. One of these resources is the notion of the quantum bit (*qubit*), which is the quantum computational extension of the standard classical bit.

A *quantum bit* or *qubit* is a pure state in the Hilbert space $C^{2}$. The standard orthonormal basis {|0〉,|1〉} of $C^{2}$, where $|0\rangle ={\left(1,0\right)}^{†}$ and $|1\rangle ={\left(0,1\right)}^{†}$, is usually called the *logical basis*. This name comes from the fact that the logical truth is related to vector |1〉 and, analogously, the falsity to the vector |0〉. Thus, a pure state |ψ〉 in $C^{2}$ can be written as a linear combination of the truth vectors $|\psi \rangle =c_{0}|0\rangle +c_{1}|1\rangle$ with $c_{0}$ and $c_{1}$ complex numbers under the condition $|c_{0}{|}^{2}+{\left|c_{1}\right|}^{2}=1$. By the celebrated Born rule, but also from a logical perspective, any qubit $|\psi \rangle =c_{0}|0\rangle +c_{1}|1\rangle$ can be considered as a piece of information, where the quantity $|c_{0}{|}^{2}$ represents the probability of the information described by the basic vector |0〉 and, analogously, $|c_{1}{|}^{2}$ corresponds to the probability of the information described by |1〉. The two basis elements |0〉 and |1〉 are usually considered as the encoding of the standard bit values 0 and 1, respectively. Hence, we focus on the qubit probability value $p(|\psi \rangle )=|c_{1}{|}^{2}$ that is related to the vector |1〉 associated with truth.

In general, quantum states considered in quantum computation lie in the tensor product ${⊗}^{n}C^{2}=C^{2}⊗C^{2}⊗\ldots ⊗C^{2}$ (*n*-times), which is a $2^{n}$-dimensional complex space. The expression $|x_{1},x_{2},\ldots ,x_{n}\rangle$ is an abbreviation of a vector in ${⊗}^{n}C^{2}$ expressed as the tensor product $|x_{1}\rangle ⊗|x_{2}\rangle ⊗\ldots ⊗|x_{n}\rangle$ where $|x_{i}\rangle \in C^{2}$. The $2^{n}$-*computational basis* of ${⊗}^{n}C^{2}$ consists of the $2^{n}$ orthogonal states |ι〉, $0\leq \iota \leq 2^{n}$ where ι is in binary representation and |ι〉 can be seen as tensor product of states, i.e., the Kronecker product, $\left|\iota \rangle =\right|\iota_{1}\rangle ⊗|\iota_{2}\rangle ⊗\ldots ⊗|\iota_{n}\rangle$, where $\iota_{j}\in \left\{0,1\right\}$. In this way, a pure state $|\psi \rangle \in {⊗}^{n}C^{2}$ can be represented as $|\psi \rangle =\sum_{\iota =1}^{2^{n}}c_{\iota }|\iota \rangle$, where $\sum_{\iota =1}^{2^{n}}{\left|c_{\iota }\right|}^{2}=1$.

In the standard models, a quantum circuit is represented by a composition of *quantum gates*, mathematically represented by *unitary operators* applied on pure states of a Hilbert space ${⊗}^{n}C^{2}$ [2]. It defines the ideal standard model for quantum computation that is mathematically based on “*qubits-unitary operators*”.

However, in the model described above, it is difficult to formally describe several relevant processes, such as measurement, decoherence and noise, playing crucial roles in quantum computing. For example, at the end of the computation, and in order to obtain the result of a computational process, a non-unitary operation, a measurement, is applied. In this way, the state can be represented as a probability distribution over different pure states, i.e., it becomes a mixed state. These facts have motivated several authors to focus on a general model of quantum computation, where mixed states are in place of pure ones [6,13,14,15,16]. In what follows, we provide a description of this model, which is better suited to our development.

Let us note that to each vector of the logical basis of $C^{2}$ can be associated two density operators $P_{1}=\left|1\rangle \langle 1\right|$ and $P_{0}=\left|0\rangle \langle 0\right|$, representing the classical truth values. Moreover, we can naturally extend these notions of truth to arbitrary Hilbert spaces of the form ${⊗}^{n}C^{2}$ by considering the following density operators:

(7) $$P_{1}^{\left(n\right)}={⊗}^{n-1}I⊗P_{1}\;\mathrm{and}\;P_{0}^{\left(n\right)}={⊗}^{n-1}I⊗P_{0}.$$

By applying the Born rule, we consider the probability of a density operator ρ in the following way:

(8) $$p\left(\rho \right)=Tr\left(P_{1}^{\left(n\right)}\rho \right).$$

By straightforward calculation, for each n∈N, we can show that

(9) $$p\left(P_{1}^{\left(n\right)}\right)=1\;\mathrm{and}\;p\left(P_{0}^{\left(n\right)}\right)=0.$$

We also note that, in the particular case where ρ=|ψ〉〈ψ|, with $|\psi \rangle =c_{0}|0\rangle +c_{1}|1\rangle$, we have that $p\left(\rho \right)=|c_{1}{|}^{2}$. Therefore, the probability value associated with ρ is the generalization of the probability value considered for qubits.

Let H be a Hilbert space. Let us denote by L(H) the space of linear operators on H. A *quantum operation* [17] is a linear operator of the form $E:L\left(H_{1}\right)\to L\left(H_{2}\right)$ such that, for each density operator, $\rho \in L(H_{1})$,

(10) $$E\left(\rho \right)=\sum_{i}A_{i}\rho A_{i}^{†}$$

where $A_{i}$ are linear operators s.t. $\sum_{i}A_{i}^{†}A_{i}=I$. It is easy to show that a quantum operation is a map from density operators to density operators. Each unitary operator *U* in L(H) comes from a quantum operation U such that, for each density operator, ρ in H

(11) $$U\left(\rho \right)=U\rho U^{†}.$$

The probability associated with a state after the application of the quantum operation U is given by:

(12) $$\begin{array}{c} p\left(U\left(\rho \right)\right)=tr\left(P_{1}^{\left(n\right)}\left(U\rho U^{†}\right)\right)=tr\left(\left(U^{†}P_{1}^{\left(n\right)}U\right)\rho \right). \end{array}$$

Thus, quantum operations are generalizations of unitary operators. This provides a powerful model for quantum computation, where irreversible processes can be also considered. This model based on density operators and quantum operations is called *quantum computation with mixed states* ([13,16]).

## 4. Logical Systems from Quantum Gates

As in classical computation, quantum computation with mixed states motivates several logical systems arising from families of quantum gates [13]. Each of these logical structures is based on the algebraic properties of a determinate family of quantum gates that mathematically describe the architecture of the quantum circuits where the information is processed. In this way, each family of quantum gates gives place to a logical system based on the compositional properties of their circuits.

As expected, these logical systems, in general, are substantially different from the classical logic. However, their notion of logical consequence is inspired by classical digital techniques:

- If *T* is a compositional circuit, we ask if an input state of *T*, represented by a string of bits 0 and 1, forces a certain output state of *T* given by a bit, which could be either 0 or 1.

It is well known that this problem is solvable by effective procedures based on classical logic. Thus, this problem can be naturally extended by considering circuits produced by assemblies of quantum gates. In order to do this, in the model of quantum computation with mixed states, both the input and the output of quantum circuits are represented by appropriate density operators, and quantum gates are represented by quantum operations, as we have introduced in Section 3. Then, by considering relations between the input and the output of the quantum circuits, it is possible to define the notions of logical consequence. These logics have a common interpretation based on the probability values introduced in Equation (8) and have been studied by several authors [4,6,14].

Formally, these logical systems, arising from a family of quantum gates, have an underlying propositional language $L_{F}\left(X\right)$, where with *X* we indicate a non-empty set of variables and with F we denote a set of connectives. Propositional variables are interpreted in a set D of density operators, and each connective f∈F is interpreted in a natural way as a quantum operation $U_{f}$ closed on D. The semantic component of this language is built up from the following notion of interpretation. An *interpretation* of $L_{F}\left(X\right)$ in D is a function $e:L_{F}\left(X\right)\to D$ such that, for each f∈F with arity *k*, $e\left(f\left(x_{1},\ldots ,x_{k}\right)\right)=U_{f}\left(e\left(x_{1}\right),\ldots ,e\left(x_{k}\right)\right)$. A notion of valuation based on the probability assignment given in Equation (8) is also introduced. Indeed, a *valuation* is a function over the unitary real interval $v:L_{F}\left(X\right)\to \left[0,1\right]$ such that *v* can be factorized in the following way: (13)
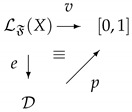


Since an interpretation always determines a valuation, for each interpretation *e*, we denote by $e_{p}$ the valuation associated with *e*. In this way, it is possible to introduce abstract notions of logical consequence ⊢ related to D in the following way:

α⊢φiff:R[v(α),v(φ)]

where $R\subseteq {\left[0,1\right]}^{2}$ is a reflexive and transitive relation. Note that the natural extension of classical logical consequence can be formulated as follows:

(14) $$\alpha ⊢\varphi \;iff:\;e_{p}\left(\alpha \right)=1\;then\;e_{p}\left(\varphi \right)=1.$$

Since the semantic component of these propositional systems assumes its truth value in the real interval [0,1], they can be framed in the family of fuzzy logics [8,9]. Moreover, these semantics are formulated in a non-Kolmogorovian probability model based on the Born rule [18]. More precisely, let us note that, in our case, the algebra of events does not have a Boolean structure. Thus, the logical systems introduced in this section can also be seen as generalizations of probabilistic logics, described by E. W. Adams [19], where the transmission of probability values thorough inferences is studied.

## 5. Quantum Fredkin Gate and Reversible Logic

The classical Fredkin gate can be framed within the so-called reversible computation. A reversible computing system [20] is such that every computational configuration, i.e., a state of the whole system, has exactly one previous configuration. Therefore, a backwards computation is performed by its inverse computing system. The research for reversible computing originated from an investigation of energy dissipation in computing systems. Power dissipation is known as the most notable limiting factor in all nano-electronic design techniques and, whenever we use logically irreversible gates, such as AND, NAND, OR, XOR, etc., a considerable amount of energy is dissipated into the environment. This loss of energy is strictly related to an information loss during computational process. In this perspective, the interest in reversible computation arises in order to reduce heat dissipation, allowing higher integration densities and also higher speed. More precisely, the mentioned interest in reversible computing process became crucial when Rolf Landauer, in 1961, found the existence of a lower theoretical limit of energy consumption in computation [21].

A reversible computing system is based on reversible logical gates represented by injective Boolean functions. In other words, logical reversibility refers to the possibility to reconstruct the input from the output of a Boolean function. One of the most important reversible gates is the Fredkin gate, proposed by Fredkin and Toffoli in [22], introduced in the following definition.

**Definition** **3.** *The Fredkin gate is defined as the reversible ternary connective $F:{\left\{0,1\right\}}^{3}\to {\left\{0,1\right\}}^{3}$ such that*

$$F\left(x,y,z\right)=(x,\;\;y\;\hat{+}\;x\left(y\;\hat{+}\;z\right),\;\;z\;\hat{+}\;x\left(y\;\hat{+}\;z\right))$$

*where $\hat{+}$ is the sum modulo 2, also called XOR.*

The truth table associated with this gate is given by

$$\begin{array}{cccccc} x & y & z & x & y\;\hat{+}\;x\left(y\;\hat{+}\;z\right) & z\;\hat{+}\;x\left(y\;\hat{+}\;z\right))\\ 0 & 0 & 0 & 0 & 0 & 0\\ 0 & 0 & 1 & 0 & 0 & 1\\ 0 & 1 & 0 & 0 & 1 & 0\\ 0 & 1 & 1 & 0 & 1 & 1\\ 1 & 0 & 0 & 1 & 0 & 0\\ 1 & 0 & 1 & 1 & 1 & 0\\ 1 & 1 & 0 & 1 & 0 & 1\\ 1 & 1 & 1 & 1 & 1 & 1 \end{array}$$

In other words, the Fredkin gate behaves as follows: the first bit *x* plays the role of a control bit, i.e., it remains unaffected by the action of the gate, and the second and the third bits, *y* and *z*, play the role of target bits, which are swapped if and only if the control bit *x* is 1 (otherwise, they do not change).

The Fredkin gate is universal for classical computation; indeed, any classical circuit can be built from an ensemble of Fredkin gates alone. Moreover, this gate can be considered a functionally complete connective for the classical propositional logic in the sense that it can be used to implement AND, OR and NOT. Indeed,

(15) $$\begin{array}{ccc} F(x,0,1) & = & \left(x,\;-\;,NOT(x)\right) \end{array}$$

(16) $$\begin{array}{ccc} F(x,y,0) & = & \left(x,\;-\;,AND(x,y)\right) \end{array}$$

(17) $$\begin{array}{ccc} F(x,y,1) & = & (x,\;OR(x,y),\;-). \end{array}$$

The Fredkin gate admits a natural extension as a quantum gate acting on qubits in the following way.

**Definition** **4.** *Let $\left|x\rangle =\right|x_{1},x_{2},\ldots ,x_{n}\rangle$, $\left|y\rangle =\right|y_{1},y_{2},\ldots ,y_{m}\rangle$ and $\left|z\rangle =\right|z_{1},z_{2}\ldots ,z_{l}\rangle$ be vectors of the standard orthonormal basis in ${⊗}^{n}C^{2}$, ${⊗}^{m}C^{2}$ and ${⊗}^{l}C^{2}$, respectively. Then, the quantum Fredkin gate is defined as follows:*

$$\begin{array}{ccc} F^{\left(n,m,l\right)}(|x\rangle ⊗|y\rangle ⊗\left|z\rangle \right) & = & \left|x\rangle ⊗\right|y_{1}\cdots y_{m-1},\;y_{m}\;\hat{+}\;x_{n}\left(y_{m}\hat{+}z_{l}\right)\rangle \\ & & ⊗|z_{1}\ldots z_{l-1},\;z_{l}\;\hat{+}\;x_{n}\left(y_{m}\hat{+}z_{l}\right)\rangle . \end{array}$$

Notice that $F^{\left(n,m,l\right)}$ is a linear operator on ${⊗}^{\left(n+m+l\right)}C^{2}$. The following proposition establishes a functional representation of the quantum Fredkin gate as a unitary matrix.

**Proposition** **1.** *For any natural number n,m,l≥1, the quantum Fredkin gate $F^{\left(n,m,l\right)}$ assumes the following matrix representation:*

$$\begin{array}{ccc} F^{\left(n,m,l\right)} & = & P_{0}^{\left(n\right)}⊗I^{\left(m+l\right)}+P_{1}^{\left(n\right)}⊗{SWAP}^{\left(m\;,\;l\right)}\\ & = & I^{\left(n-1\right)}⊗\left(P_{0}⊗I^{\left(m+l\right)}+P_{1}⊗SWAP^{\left(m,l\right)}\right)\\ & = & I^{\left(n-1\right)}⊗\left[\begin{array}{cc} I^{\left(m+l\right)} & 0\\ 0 & SWAP^{\left(m,l\right)} \end{array}\right] \end{array}$$

*where*

$$\begin{array}{ccc} {SWAP}^{\left(m\;,\;l\right)} & = & I^{\left(m-1\right)}⊗\left[\begin{array}{cc} P_{0}^{\left(l\right)} & L_{1}^{\left(l\right)}\\ L_{0}^{\left(l\right)} & P_{1}^{\left(l\right)} \end{array}\right]\\ & = & P_{0}^{\left(m\right)}⊗P_{0}^{\left(l\right)}+P_{1}^{\left(m\right)}⊗P_{1}^{\left(l\right)}+L_{0}^{\left(m\right)}⊗L_{1}^{\left(l\right)}+L_{1}^{\left(m\right)}⊗L_{0}^{\left(l\right)},\\ L_{0}^{\left(l\right)} & = & I^{\left(l-1\right)}⊗|0\rangle \langle 1|,\\ L_{1}^{\left(l\right)} & = & I^{\left(l-1\right)}⊗|1\rangle \langle 0|. \end{array}$$

**Proof.**  See ([23], Proposition 5.1). □

In what follows, we denote by $D_{n}$ the set of density operators in ${⊗}^{n}C^{2}$. Since $F^{\left(n,m,l\right)}$ is a unitary operator satisfying $F^{\left(n,m,l\right)}={F^{\left(n,m,l\right)}}^{†}$, by Equation (11), we can extend $F^{\left(n,m,l\right)}$ to a quantum operation acting on density operators of ${⊗}^{\left(n+m+l\right)}C^{2}$ as follows:

(18) $$F^{\left(n,m,l\right)}\left(\rho \right)=F^{\left(n,m,l\right)}\rho F^{\left(n,m,l\right)}.$$

By Equation (12), the probability assigned to F(ρ), i.e., its truth function, is given by

(19) $$p\left(F^{\left(n,m,l\right)}\left(\rho \right)\right)=Tr\left(F^{\left(n,m,l\right)}\cdot P_{1}^{\left(n+m+l\right)}\cdot F^{\left(n,m,l\right)}\rho \right).$$

Thus, $F^{\left(n,m,l\right)}$ is the representation of the Fredkin gate in the model of quantum computation with mixed states.

The following proposition establishes the probability value of a state after the application of $F^{\left(n,m,l\right)}$ over a 3-factorizable quantum state. In this particular case, the condition of the ternary connective of the classical Fredkin gate is preserved in this quantum computational framework.

**Proposition** **2.** *Let us consider the factorized density operator $\rho =\rho_{n}⊗\rho_{m}⊗\rho_{l}$ where $\rho_{i}\in D_{i}$ for i∈{n,m,l}. Then,*

$$p(F^{\left(n,m,l\right)}\left(\rho_{n}⊗\rho_{m}⊗\rho_{l}\right)=\neg_{{}_{L}}p\left(\rho_{n}\right)\cdot p\left(\rho_{l}\right)⊕p\left(\rho_{n}\right)\cdot p\left(\rho_{m}\right)$$

*where*

$$\begin{array}{ccc} \neg_{{}_{L}}x & = & 1-x,\;Łukasiewicz\;negation\\ x⊕y & = & \min\{x+y,1\}\;Łukasiewicz\;sum\;or\;disjunction\\ x\cdot y & = & xy\;Product\;t-norm. \end{array}$$

**Proof.**  See ([23], Proposition 6.1). □

The above proposition establishes a deep relation between quantum circuits built from the quantum Fredkin gates and the fuzzy logic mentioned in Section 4. Indeed, the operations $\left\{\neg_{{}_{Ł}},⊕,\cdot \right\}$ in the real interval [0,1] define the standard model of a fuzzy system called *Product Łukasiewicz logic* or *Product MV-logic* [24,25], which is an expansion of the infinite-valued Lucasiewicz calculus [8].

## 6. Extending the Propositional Classical Logic via Quantum Fredkin Compositional Logic

By *quantum Fredkin compositional logic*, we refer to the logical expressions representing compositional quantum circuits built from the quantum Fredkin. In this framework, we provide logical expressions representing circuits built from configurations of the quantum Fredkin gate able to extend the basic connectives of the classical logic.

A natural extension of the basic connectives of the classical logic can be obtained by generalizing the classical Fredkin gate expression of the functionally complete set of connectives {¬,∧} given in Equations (16) and (17). In this way, we can consider the following instances of the $F^{\left(n,m,1\right)}$:

(20) $$\begin{array}{ccc} \mathrm{NOT}(\rho ) & = & F^{\left(n,1,1\right)}\left(\rho ⊗P_{0}⊗P_{1}\right)\;\mathrm{if}\rho \in D_{n}, \end{array}$$

(21) $$\begin{array}{ccc} \mathrm{AND}(\rho ⊗\sigma ) & = & F^{\left(n,m,1\right)}\left(\rho ⊗\sigma ⊗P_{0}\right)\;\mathrm{if}\rho \in D_{n},\sigma \in D_{m}. \end{array}$$

The following proposition shows the semantic interpretation of NOT and AND via the probability value introduced in Equation (8).

**Proposition** **3.** *Let $\rho \in D_{n}$, $\sigma \in D_{m}$. Then,*

*1.*  *$p\left(\mathrm{NOT}\left(\rho \right)\right)=\neg_{{}_{L}}p\left(\rho \right)=1-p\left(\rho \right)$,*
*2.*  *p(AND(ρ⊗σ))=p(ρ)·p(σ)=p(ρ)p(σ)*.

**Proof.**  By Equation (9) we have that $p(P_{1})=1$ and $p(P_{0})=0$. Let $\rho \in D_{n}$ and $\sigma \in D_{m}$. Then, by Proposition 2,

$$\begin{array}{ccc} p(\mathrm{NOT}(\rho )) & = & p(F^{\left(n,1,1\right)}\left(\rho ⊗P_{0}⊗P_{1}\right)),\\ & = & \neg_{{}_{L}}p\left(\rho \right)\cdot p\left(P_{1}\right)⊕p\left(\rho \right)\cdot p\left(P_{0}^{\left(m\right)}\right)\\ & = & \neg_{{}_{L}}p\left(\rho \right). \end{array}$$

$$\begin{array}{ccc} p(\mathrm{AND}(\rho ⊗\sigma )) & = & p(F^{\left(n,m,1\right)}\left(\rho ⊗\sigma ⊗P_{0}\right))\\ & = & \neg_{{}_{L}}p\left(\rho \right)\cdot p\left(P_{0}\right)⊕p\left(\rho \right)\cdot p\left(\sigma \right)\\ & = & 0+p(\rho )\cdot p(\sigma )=p(\rho )\cdot p(\sigma ). \end{array}$$

□

**Figure 1:**
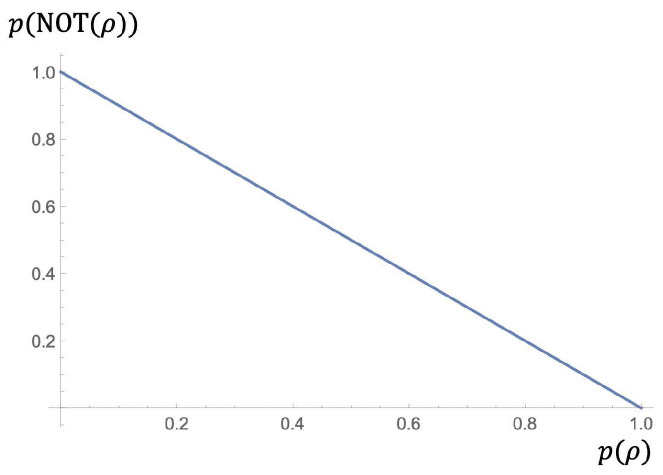
Fuzzy behavior of NOT.

**Figure 2:**
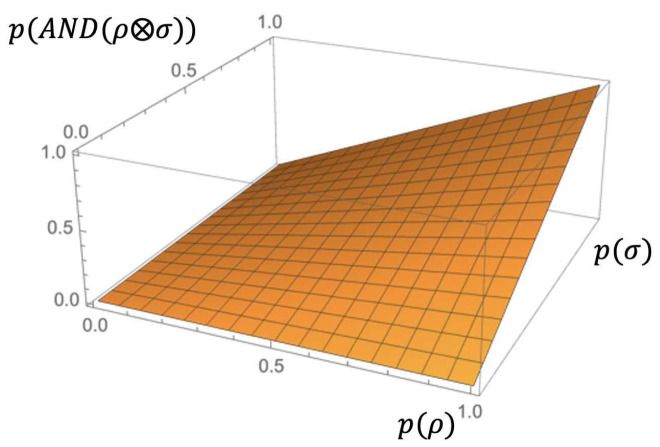
Fuzzy behavior of AND.

In the following pictures we can see the fuzzy behavior of NOT (Figure 1) and AND (Figure 2), respectively.

By the above proposition, the system {NOT,AND} can be identified to the reduct $\left\{\neg_{{}_{Ł}},\cdot \right\}$ of the fuzzy system given by the Product Lucasiewicz logic mentioned at the end of Section 5. We also note that if we restrict {NOT,AND} to $P_{0}^{\left(n\right)}$ and $P_{1}^{\left(n\right)}$, these quantum gates play the roles of the classical negation and conjunction, respectively. In this way, {NOT,AND} represent a genuine extension of the classical logic.

In classical logic, by the functional completeness of the set {¬,∧}, the concepts of contradiction and tautology can be syntactically represented by involving these connectives. Indeed, contradictions are those formulas equivalent to x∧¬x and tautologies are those formulas equivalent to ¬(x∧¬x). Thus, the formula x∧¬x is referred to as *syntactic contradiction* and the formula ¬(x∧¬x) is referred to as *syntactic tautology*. In the extension {NOT,AND}, the mentioned syntactic representation for contradictions and tautologies is lost. This is because the set of real numbers does not contain zero divisors. Therefore, we can say that there is not an algebraic expression built from $\left\{\neg_{L},\cdot \right\}$ able to produce the constant functions 1 or 0. However, the {AND,NOT} extensions of the syntactic contradiction and the syntactic tautology show some interesting fuzzy properties. The natural extensions of the syntactic contradiction and the syntactic tautology to the system {AND,NOT} are the following:

(22) $$\begin{array}{ccc} x\wedge \neg x & ⟹ & \mathrm{AND}(\rho ⊗\mathrm{NOT}(\rho ))\;\left[\mathrm{syntactic contradiction}\right]. \end{array}$$

(23) $$\begin{array}{ccc} \neg (x\wedge \neg x) & ⟹ & \mathrm{NOT}(\mathrm{AND}(\rho ⊗\mathrm{NOT}(\rho )))\;\left[\mathrm{syntactic tautology}\right]. \end{array}$$

By Proposition 3, we can see that

(24) $$\begin{array}{ccc} p\left(\mathrm{AND}(\rho ⊗\mathrm{NOT}(\rho ))\right) & = & p(\rho )(1-p(\rho )), \end{array}$$

(25) $$\begin{array}{ccc} p\left(\mathrm{NOT}(\mathrm{AND}(\rho ⊗\mathrm{NOT}(\rho )))\right) & = & 1-p(\rho )(1-p(\rho )). \end{array}$$

**Figure 3:**
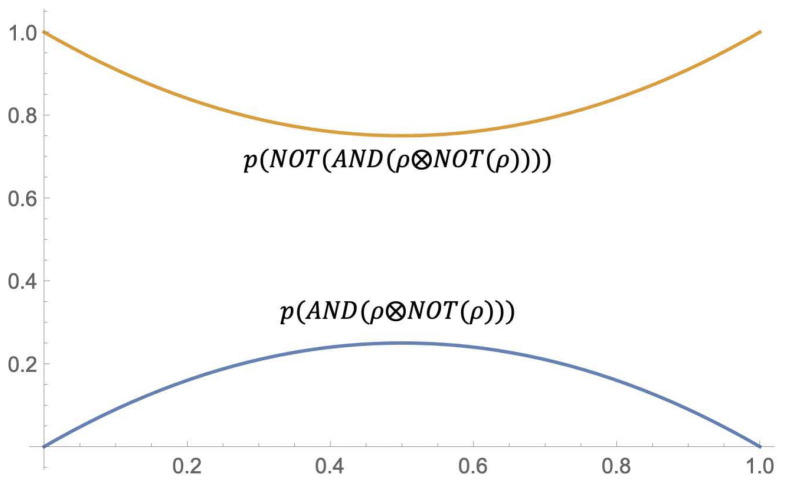
Fuzzy extension of tautology and contradiction.

The following graphics (Figure 3) show the semantic behavior of the {AND,NOT} extensions of the syntactic contradiction, represented by the blue curve, and the syntactic tautology, represented by the brown curve.

By simple calculus, we can show that

(26) $$0\leq p\left(\mathrm{AND}(\rho ⊗\mathrm{NOT}(\rho ))\right)\;\leq \;\frac{1}{4},$$

(27) $$\frac{3}{4}\leq p\left(\mathrm{NOT}(\mathrm{AND}(\rho ⊗\mathrm{NOT}(\rho )))\right)\;\leq \;1.$$

We can also see that the extension of the syntactic contradiction cannot ever reach 1, the extension of the syntactic tautology cannot ever reach 0, and, in both cases, there is a classical behavior over $P_{0}^{\left(n\right)}$ and $P_{1}^{\left(n\right)}$, as expected.

In propositional classical logic, the notion of logical consequence, in the case of finite set formulas, is related to a tautological conditional. More precisely, we say that a formula ψ is a *logical consequence* of φ if the conditional φ→ψ is a tautology. Note that φ→ψ can be equivalently expressed as ¬(φ∧¬ψ). The last formula allows us to extend this notion of logical consequence to the system {NOT,AND} by considering the extended conditional

(28) $$\mathrm{NOT}\left(\mathrm{AND}(\rho ⊗\mathrm{NOT}(\sigma )\right).$$

By Proposition 3, we can see that

(29) $$p\left(\mathrm{NOT}\left(\mathrm{AND}(\rho ⊗\mathrm{NOT}(\sigma )\right)\right)=1-p\left(\rho \right)+p\left(\rho \right)p\left(\sigma \right).$$

Thus, following the classical notion of logical consequence, we can say that the state σ is a logical consequence modulo Fredkin of the state ρ if 1−p(ρ)+p(ρ)p(σ)=1, which is equivalent to: p(σ)=1 or p(ρ)=0.

## 7. Holistic Extension for the Classical Logic via Quantum Fredkin Gate

The holistic characteristic related to non-factorizable bipartite quantum states, formally described in Section 2, is a crucial tool in order to establish an holistic-type extension of the classical logic. More precisely, the mentioned holistic extension is based on the behavior of the quantum Fredkin gates over bipartite quantum states.

The first relevant fact in this holistic extension is that, when we consider quantum Fredkin gates acting over the full family of bipartite quantum states, the arity of the connectives set {NOT,AND} is not determinate. Indeed, the syntax of the usual logical languages is regulated by strict rules formulated in terms of a recursive procedure. In this process, the notion of the formula is defined by starting with a primitive notion of formulas, called atomic formulas, represented by propositional variables or constants. Then, complex formulas are obtained recursively from atomic propositions that are assembled by connectives. For each connective, a fixed natural number, the *arity*, is assigned. The arity fixes the number of formulas that the connectives assemble. When we consider an algebraic semantic for these logical systems, then a *n*-ary connective is considered as an algebraic operation with *n* arguments. Thus, the arity is an invariant property associated with a connective. All these ideas were already taken into account for the system {NOT,AND} when factorizable states were considered. More precisely, AND has been considered as a binary connective acting on an ideal factorizable state of the form $\rho_{n}⊗\rho_{m}$.

However, this is not the case. For example, quantum systems frequently interact with the environment, creating correlations. Then, for a more realistic approach, we can assume that the input of the AND can also be a non-factorizable mixed state. In this general case, AND can be seen as a unary operator. This particular behavior of AND over non-factorizable states motivates an holistic-type extension of classical conjunction. In order to describe the action of AND over non-factorizable states, we first establish some technical results regarding the quantum Fredkin gate.

Let ρ be a density operator acting on ${⊗}^{n+m}C^{2}$ and let us denote by $r_{i}$ the *i*-th diagonal element of ρ, with $1\leq i\leq 2^{n+m}$. Let us consider a partition of the diagonal of ρ in $2^{n}$ blocks, each one containing $2^{m}$ elements:

$$diag\left(\rho \right)=[\left(r_{1},\cdots ,r_{2^{m}}\right),\left(r_{2^{m}+1},\cdots ,r_{2^{m+1}}\right),\cdots ,\left(r_{\left(2^{n}-1\right)2^{m}+1},\cdots ,r_{2^{m+n}}\right)].$$

Denoting by $B_{i}$ (with $1\leq i\leq 2^{n}$) the *i*-th block containing $2^{m}$ elements of diag(ρ), we write

$$diag\left(\rho \right)=\left[B_{1},B_{2},\cdots ,B_{2^{n}}\right].$$

Based on diag(ρ), we also consider the following parameters:

- $\alpha^{n,m}\left(\rho \right)=\sum_{i=1}^{2^{n-1}}\sum_{j=1}^{2^{m-1}}r_{\left(2i-1\right)2^{m}+2j}$, i.e., the sum of the even diagonal elements of the even blocks of diag(ρ),
- $\beta^{n,m}\left(\rho \right)=\sum_{i=1}^{2^{n-1}}\sum_{j=1}^{2^{m-1}}r_{\left(2i-1\right)2^{m}+2\left(j-1\right)}$, i.e., the sum of the odd diagonal elements of the even blocks of diag(ρ),
- $\gamma^{n,m}\left(\rho \right)=\sum_{i=1}^{2^{n-1}}\sum_{j=1}^{2^{m-1}}r_{\left(2i-2\right)2^{m}+2j}$, i.e., the sum of the even diagonal elements of the odd blocks of diag(ρ).

By Definition 2, it is straightforward to see the following result.

**Proposition** **4.** *Let ρ be a density operator on ${⊗}^{n+m}C^{2}$ and let $\rho_{n}=tr_{{⊗}^{m}C^{2}}\left(\rho \right)$ and $\rho_{m}=tr_{{⊗}^{n}C^{2}}\left(\rho \right)$ the reduced states of ρ. Then,*

*1.*  *$p\left(\rho_{n}\right)=\alpha^{n,m}\left(\rho \right)+\beta^{n,m}\left(\rho \right)$;*
*2.*  *$p\left(\rho_{m}\right)=\alpha^{n,m}\left(\rho \right)+\gamma^{n,m}\left(\rho \right)=p\left(\rho \right)$.*

By Equation (6), any density operator ρ on ${⊗}^{n+m}C^{2}$ can be decomposed as $\rho =\rho_{n}⊗\rho_{m}+M\left(\rho \right)$, where $\rho_{n}$ and $\rho_{m}$ are the reduced states of ρ with respect to the subsystems ${⊗}^{m}C^{2}$ and ${⊗}^{n}C^{2}$, respectively, and M(ρ) is the holistic component whose trace is null. Therefore, M(ρ) has no influence on the probability value of p(ρ).

**Proposition** **5.** *Let ρ be a density operator on ${⊗}^{n+m}C^{2}$. Then, we have that:*

$$p\left(F^{\left(n,m,1\right)}\left(\rho ⊗P_{0}\right)\right)=\alpha^{n,m}\left(\rho \right).$$

**Proof.**  First, let us consider the matrix form of

$$\begin{array}{ccc} \rho ⊗P_{0} & = & \left[\begin{array}{ccccc} r_{1}⊗P_{0} & \ldots & & & \\ \vdots & r_{2}⊗P_{0} & & & \\ & & & \ddots & \\ & & & & r_{2^{n+m}}⊗P_{0} \end{array}\right]. \end{array}$$

Hence, the diagonal entries of $\rho ⊗P_{0}$ are given by

$$diag\left(\rho ⊗P_{0}\right)=r_{1},0,r_{2},0,\cdots ,r_{2^{n+m}-1},0,r_{2^{n+m}}.$$

The $diag(\rho ⊗P_{0})$ can be partitioned into $2^{n}$ blocks with length $2^{m+1}$, as follows:

$$\begin{array}{ccc} diag(\rho ⊗P_{0}) & = & [r_{1},0,r_{2},0,\cdots ,r_{2^{m}},0],\\ & & [r_{2^{m}+1},0,r_{2^{m}+2},0,\cdots ,r_{2^{m+1}},0],\cdots ,\\ & & \cdots ,[r_{\left(2^{n}-1\right)2^{m}+1},0,\cdots ,r_{2^{m+n}},0]. \end{array}$$

$$\begin{array}{ccc} F^{\left(n,m,1\right)} & = & P_{0}^{\left(n\right)}⊗I^{\left(m+1\right)}+P_{1}^{\left(n\right)}⊗Swap^{\left(m,1\right)}\\ & = & \left[\begin{array}{ccccc} I^{\left(m+1\right)} & \ldots & & & \\ \vdots & Swap^{\left(m,1\right)} & & & \\ & & I^{\left(m+1\right)} & & \\ & & & \ddots & \\ & & & & Swap^{\left(m,1\right)} \end{array}\right]. \end{array}$$

Hence, the application of $F^{\left(n,m,1\right)}$ to $\rho ⊗P_{0}$, corresponds to applying the identity $I^{\left(m+1\right)}$ to the odd diagonal blocks of $\rho ⊗P_{0}$ and the $Swap^{\left(m,1\right)}$ to the even diagonal blocks of $\rho ⊗P_{0}$, respectively.

Let us consider a density operator σ on ${⊗}^{m+1}C^{2}$. Let $diag\left(\sigma \right)=s_{1},s_{2},\cdots ,s_{2^{m+1}}$. It is possible to divide diag(σ) into $2^{m-1}$ blocks with length 4 as follows:

$$diag\left(\sigma \right)=\left[s_{1},s_{2},s_{3},s_{4}\right],\cdots ,\left[s_{2^{m+1}-3},s_{2^{m+1}-2},s_{2^{m+1}-1},s_{2^{m+1}}\right].$$

It is easy to see that

$$\begin{array}{ccc} diag(Swap^{\left(m,1\right)}\sigma Swap^{\left(m,1\right)}) & = & [s_{1},s_{3},s_{2},s_{4}],\cdots ,\\ & & \cdots ,[s_{2^{m+1}-3},s_{2^{m+1}-1},s_{2^{m+1}-2},s^{2^{m+1}}]. \end{array}$$

In other words, the extremes of each block are left unchanged while the intermediate elements are swapped. Therefore, we have that:

$$\begin{array}{ccc} diag(F^{\left(n,m,1\right)}\left(\rho ⊗P_{0}\right)) & = & [r_{1},0,r_{2},0,\cdots ,r_{2^{m}},0],\\ & & [r_{2^{m}+1},r_{2^{m}+2},0,0,r_{2^{m}+3},r_{2^{m}+4},0,0,\cdots ,\\ & & 0,0,r_{2^{m+1}-1},r_{2^{m+1}}],\cdots ,\\ & & \cdots ,[r_{\left(2^{n}-1\right)2^{m}+1},r_{\left(2^{n}-1\right)2^{m}+2},0,0,\cdots ,\\ & & 0,0,r_{2^{m+n}-1},r_{2^{m+n}}]. \end{array}$$

Finally, $p\left(F^{\left(n,m,1\right)}\left(\rho ⊗P_{0}\right)\right)=Tr\left(P_{1}^{\left(n+m+1\right)}F^{\left(n,m,1\right)}\left(\rho ⊗P_{0}\right)\right)$, which corresponds to selecting only the even entries of $diag(F^{n,m,1}\left(\rho ⊗P_{0}\right))$. However, all the even entries of the odd blocks of $diag(F^{n,m,1}\left(\rho ⊗P_{0}\right))$ are null and the even entries of the even blocks of $diag(F^{n,m,1}\left(\rho ⊗P_{0}\right))$ correspond to the even entries of the even blocks of ρ. Hence, our claim is supported. □

**Theorem** **1.** *For any density operators ρ on ${⊗}^{n+m}C^{2}$, we have that:*

$$p\left(F^{\left(n,m,1\right)}\left(\rho ⊗P_{0}\right)\right)=p\left(\rho_{n}\right)\cdot p\left(\rho_{m}\right)+\alpha^{n,m}\left(\rho \right)\cdot \left(1+p\left(\rho \right)\right)+\beta^{n,m}\left(\rho \right)\cdot p\left(\rho \right)$$

*where $\rho_{n}$ and $\rho_{m}$ are the reduced states of ρ with respect to the subsystems ${⊗}^{m}C^{2}$ and ${⊗}^{n}C^{2}$, respectively.*

**Proof.**  By Equation (6), the state $\rho \in {⊗}^{n+m}C^{2}$ can be written as $\rho =\rho_{n}⊗\rho_{m}+M\left(\rho \right)$. Then, by the linearity of the trace and by Proposition 2, we have:

$$\begin{array}{ccc} p(F^{\left(n,m,1\right)}\left(\rho ⊗P_{0}\right)) & = & p\left(F^{\left(n,m,1\right)}\left(\left(\rho_{n}⊗\rho_{m}+M\left(\rho \right)\right)⊗P_{0}\right)\right)\\ & = & p\left(F^{\left(n,m,1\right)}\left(\rho_{n}⊗\rho_{m}⊗P_{0}\right)\right)+\\ & & p\left(F^{\left(n,m,1\right)}\left(M\left(\rho \right)⊗P_{0}\right)\right)\\ & = & p\left(\rho_{n}\right)\cdot p\left(\rho_{m}\right)+p\left(F^{\left(n,m,1\right)}\left(M\left(\rho \right)⊗P_{0}\right)\right). \end{array}$$

Thus, by Proposition 5 and Equation (6), we have that

$$\begin{array}{ccc} p\left(F^{\left(n,m,1\right)}\left(M\left(\rho \right)⊗P_{0}\right)\right) & = & p\left(F^{\left(n,m,1\right)}\left(\rho ⊗P_{0}\right)\right)-p\left(\rho_{n}\right)\cdot p\left(\rho_{m}\right)\\ & = & \alpha^{n,m}\left(\rho \right)-p\left(\rho \right)\left(\alpha^{n,m}\left(\rho \right)+\beta^{n,m}\left(\rho \right)\right)\\ & = & \alpha^{n,m}\left(\rho \right)\cdot \left(1+p\left(\rho \right)\right)+\beta^{n,m}\left(\rho \right)\cdot p\left(\rho \right). \end{array}$$

Hence, our claim is supported. □

In order to define an holistic extension of the classical conjunction from the quantum Fredkin gate, we must address the following situation: if ρ is a density operator on ${⊗}^{k}C^{2}$, where $k=n+m=n^{\prime }+m^{\prime }$ and $n\neq n^{\prime },m\neq m^{\prime }$, we generally have that $F^{\left(n,m,1\right)}\left(\rho ⊗P_{0}\right))\neq F^{\left(n^{\prime },m^{\prime },1\right)}\left(\rho ⊗P_{0}\right))$. In other words, a logical connective based on AND also requires precise information about the holistic representation of the argument in the sense of Equation (6). For this reason, we introduce the following notions:

- $\rho_{\langle n,m\rangle }$ indicates that ρ is a density operator in ${⊗}^{n+m}C^{2}$, where the holistic representation, given by Equation (6), $\rho =\rho_{n}⊗\rho_{m}+M\left(\rho \right)$ is chosen.

Thus, if we define the set

(30) $$D_{Hol}=\left\{\rho_{\langle n,m\rangle }:m,k\in N\right\}$$

then $D_{Hol}$ is the universe where the holistic extension defines their operations. The candidate to define the holistic extension of the conjunction is

$${\mathrm{AND}}_{H}\left(\rho_{\langle n,m\rangle }\right)=F^{\left(n,m,1\right)}\left(\rho ⊗P_{0}\right)).$$

However, this operation is not well-defined as a closed operation in $D_{Hol}$. Indeed, we need to fix an holistic representation for the image of $\rho_{\langle n,m\rangle }$, given by the value ${\mathrm{AND}}_{H}\left(\rho_{\langle n,m\rangle }\right)$, so that the operation ${\mathrm{AND}}_{H}$ is closed.

In order to do this, we first note that, by Proposition 1, $F^{\left(n,m,1\right)}$ can be rephrased as follows:

$$\begin{array}{ccc} F^{\left(n,m,1\right)} & = & \left(P_{0}^{\left(n\right)}⊗I^{\left(m\right)}\right)⊗I+P_{1}^{\left(n\right)}⊗{SWAP}^{\left(m\;,\;l\right)}\\ & = & \left(P_{0}^{\left(n\right)}⊗I^{\left(m\right)}\right)⊗I+(\left(P_{1}^{\left(n\right)}⊗P_{0}^{\left(m\right)}\right)⊗P_{0}+\\ & & \left(P_{1}^{\left(n\right)}⊗P_{1}^{\left(m\right)}\right)⊗P_{1}+\left(P_{1}^{\left(n\right)}⊗L_{0}^{\left(m\right)}\right)⊗L_{1}+\\ & & \left(P_{1}^{\left(n\right)}⊗L_{1}^{\left(m\right)}\right)⊗L_{0}). \end{array}$$

Then, by the above expression and the basic properties of the tensor product, $F^{\left(n,m,1\right)}\left(\rho ⊗P_{0}\right))$ can be written as

$$\begin{array}{ccc} F^{\left(n,m,1\right)}\left(\rho ⊗P_{0}\right) & = & F^{\left(n,m,1\right)}\left(\rho ⊗P_{0}\right))F^{\left(n,m,1\right)}\\ & = & \sum_{i}\left(A_{i}^{n+m}⊗A_{i}\right)\left(\rho ⊗P_{0}\right)\left(A_{i}^{n+m}⊗A_{i}\right)\\ & = & \sum_{i}\left(A_{i}^{n+m}\rho A_{i}^{n+m}\right)⊗\left(A_{i}P_{0}A_{i}\right) \end{array}$$

where, for each *i*, $A_{i}^{n+m}$ is an operator on ${⊗}^{n+m}C^{2}$ and $A_{i}$ is an operator on $C^{2}$. The last expression of $F^{\left(n,m,1\right)}\left(\rho ⊗P_{0}\right))$ suggests a privileged holistic representation for the codomain $F^{\left(n,m,1\right)}$ given by $F^{\left(n,m,1\right)}\left(\rho ⊗P_{0}\right){)}_{\langle n+m,1\rangle }$. Thus, we can introduce an holistic-type extension for the classical logic in the following way.

**Definition** **5.** *The holistic extension for the classical logic based on the Fredkin gate is given by the pair of connectives $\left\{{\mathrm{NOT}}_{H},{\mathrm{AND}}_{H}\right\}$ acting on $D_{Hol}$, where*

$$\begin{array}{ccc} {\mathrm{NOT}}_{H}\left(\rho_{\langle n,m\rangle }\right) & = & F^{\left(n,1,1\right)}\left(\rho ⊗P_{0}⊗P_{1}\right){)}_{\langle n+m+1,1\rangle }\\ {\mathrm{AND}}_{H}\left(\rho_{\langle n,m\rangle }\right) & = & F^{\left(n,m,1\right)}\left(\rho ⊗P_{0}\right){)}_{\langle n+m,1\rangle }. \end{array}$$

The following proposition describes the relation between the truth functional behavior of the system {NOT,AND} and the holistic system $\left\{{\mathrm{NOT}}_{H},{\mathrm{AND}}_{H}\right\}$.

**Proposition** **6.** *Let ρ be a density operator on ${⊗}^{n+m}C^{2}$ and let $\rho_{n}=tr_{{⊗}^{m}C^{2}}\left(\rho \right)$ and $\rho_{m}=tr_{{⊗}^{n}C^{2}}\left(\rho \right)$ the reduced states of ρ. Then,*

*1.*  *$p\left({\mathrm{NOT}}_{H}\left(\rho_{\langle n,m\rangle }\right)\right)=p\left(\mathrm{NOT}(\rho )\right)$,*
*2.*  *$p\left({\mathrm{AND}}_{H}\left(\rho_{\langle n,m\rangle }\right)\right)=p\left(\mathrm{AND}(\rho_{n}⊗\rho_{m})\right)+\alpha^{n,m}\left(\rho \right)\cdot \left(1+p\left(\rho \right)\right)+\beta^{n,m}\left(\rho \right)\cdot p\left(\rho \right)$.*

**Proof.**  Follows from Proposition 3 and Theorem 1. □

As expected, since NOT acts as a unary connective, from a truth functional point of view, its holistic extension ${\mathrm{NOT}}_{H}$ is the same. In the case of ${\mathrm{AND}}_{H}$, its truth functional behavior is given by the fuzzy behavior of AND over the partial traces of a state plus an holistic component. Let us also remark that while classical logic needs at least one binary connective to describe any possible truth function, $\left\{{\mathrm{NOT}}_{H},{\mathrm{AND}}_{H}\right\}$ can describe any possible classical truth function by involving two unary connectives.

In the following example, we show the behavior ${\mathrm{AND}}_{H}$ applied to non-factorized states.

**Example** **1.** *Let us consider the following family of density operators $\rho_{abc}$ in ${⊗}^{2}C^{2}$, called *three-parameter qubit states* [26]:*

$$\begin{array}{ccc} \rho_{abc} & = & \frac{1}{4}\left[\begin{array}{cccc} 1+a & 0 & 0 & 0\\ 0 & 1-b & ic & 0\\ 0 & -ic & 1+b & 0\\ 0 & 0 & 0 & 1-a \end{array}\right], \end{array}$$

*where a,b,c are real parameters such that $a^{2}\leq 1$ and $b^{2}+c^{2}\leq 1.$ It can be proven that $\rho_{abc}$ represents a separable state if and only if $a^{2}+c^{2}\leq 1$. For each state $\rho_{abc}$, let us consider the partial traces $\rho_{abc_{1}}$, $\rho_{abc_{2}}$ and the holistic representation*

$${\rho_{abc}}_{\langle 1,1\rangle }=\rho_{abc_{1}}⊗\rho_{abc_{2}}+M\left(\rho_{abc}\right).$$

*Then, by a straightforward calculation and by Theorem 1, we can show that*

*1.*  *$p\left({\mathrm{AND}}_{H}\left({\rho_{abc}}_{\langle 1,1\rangle }\right)\right)=\frac{1-a}{4}$,*
*2.*  *$p\left(\mathrm{AND}(\rho_{abc_{1}}⊗\rho_{abc_{2}})\right)=p\left(\rho_{abc_{1}}\right)p\left(\rho_{abc_{2}}\right)=\frac{{\left(a-2\right)}^{2}-b^{2}}{16}$,*
*3.*  *$p\left(F^{1,1,1}\left(M\left(\rho_{abc}\right)⊗P_{0}\right)\right)=\frac{b^{2}-a^{2}}{16}$.*

**Figure 4:**
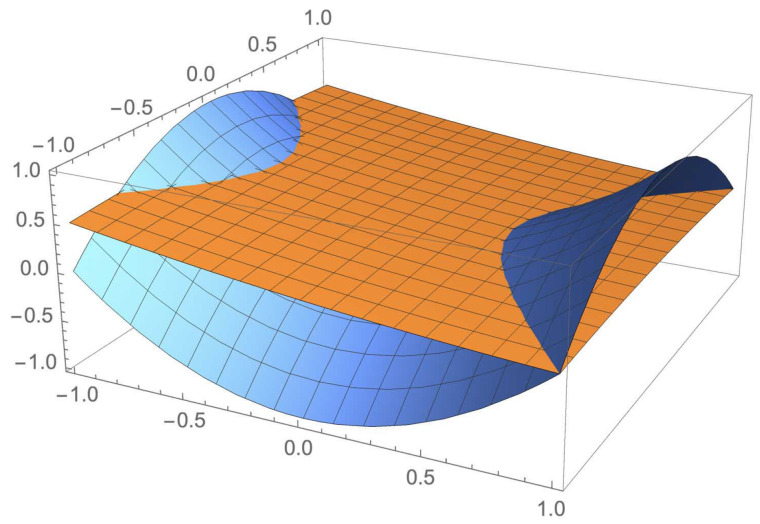
Holistic conjunction on ${\rho_{abc}}_{\langle 1,1\rangle }$.

*In Figure 4, the brown surface represents the probability inducted by the partial traces of ${\rho_{abc}}_{\langle 1,1\rangle }$ (given by the above item 2), while the blue surface represents the holistic contribution to the probability of ${\rho_{abc}}_{\langle 1,1\rangle }$ (given by the above item 3).*

## 8. An Holistic Extension for the Contradiction

The logical system $\left\{{\mathrm{NOT}}_{H},{\mathrm{AND}}_{H}\right\}$ is defined by unary connectives only. This fact does not allow us to naturally extend the syntactic representation of the classical contradiction given by x∧¬x. However, we can characterize a sub-class of $D_{Hol}$ that preserves the notion of syntactic contradiction when ${\mathrm{AND}}_{Hol}$ takes arguments on this class. Indeed, by Equation (22), the notion of syntactic contradiction extended to the fuzzy type system {NOT,AND} is given by AND(ρ,NOT(ρ)), where p(AND(ρ,NOT(ρ)))=p(ρ)(1−p(ρ)). Motivated by this idea, we first introduce the following set:

(31) $$D_{Hol}^{cont}=\left\{\rho_{\langle n,m\rangle }\in D_{Hol}:p\left(\rho_{n}\right)=1-p\left(\rho_{m}\right)\right\}.$$

**Proposition** **7.** *Let $\rho_{\langle n,m\rangle }\in D_{Hol}$. Then, the following sentences are equivalent:*

*1.*  *$\rho_{\langle n,m\rangle }\in D_{Hol}^{cont}$,*
*2.*  *$2\alpha^{n,m}\left(\rho \right)+\beta^{n,m}\left(\rho \right)+\gamma^{n,m}\left(\rho \right)=1$.*

**Proof.**  Follows immediately from Proposition 2. □

**Example** **2.** *Let us consider the subfamily of three-parameter qubit states, introduced in Example 1, given by $\rho_{1bc}$, which is*

$$\begin{array}{ccc} \rho_{1bc} & = & \frac{1}{4}\left[\begin{array}{cccc} 2 & 0 & 0 & 0\\ 0 & 1-b & ic & 0\\ 0 & -ic & 1+b & 0\\ 0 & 0 & 0 & 0 \end{array}\right], \end{array}$$

*if we consider the holistic representation ${\rho_{1bc}}_{\langle 1,1\rangle }$ Then, by straightforward calculation, we can see that*

$$2\alpha^{n,m}\left(\rho_{1bc}\right)+\beta^{n,m}\left(\rho_{1bc}\right)+\gamma^{n,m}\left(\rho_{1bc}\right)=\frac{1}{4}\left(2+\left(1+b\right)+\left(1-b\right)\right)=1.$$

*Thus, the subfamily of three-parameter qubit states of the form ${\rho_{1bc}}_{\langle 1,1\rangle }$ are elements of $D_{Hol}^{cont}$.*

The elements of $D_{Hol}^{cont}$ allow us to extend the notion of syntactic contradiction in the system $\left\{{\mathrm{NOT}}_{H},{\mathrm{AND}}_{H}\right\}$. Indeed, if $\rho_{\langle n,m\rangle }\in D_{Hol}^{cont}$, then, by Proposition 6, we have that:

$$\begin{array}{ccc} p\left({\mathrm{AND}}_{H}\left(\rho_{\langle n,m\rangle }\right)\right) & = & p\left(\mathrm{AND}(\rho_{n}⊗\rho_{m})\right)+\alpha^{n,m}\left(\rho \right)\cdot \left(1+p\left(\rho \right)\right)+\beta^{n,m}\left(\rho \right)\cdot p\left(\rho \right)\\ & = & p\left(\rho_{n}\right)\left(1-p\left(\rho_{n}\right)\right)+\alpha^{n,m}\left(\rho \right)\cdot \left(1+p\left(\rho \right)\right)+\beta^{n,m}\left(\rho \right)\cdot p\left(\rho \right). \end{array}$$

Thus, if $\rho_{\langle n,m\rangle }$ is factorizable as $\rho_{\langle n,m\rangle }=\rho_{n}⊗\rho_{m}$, then $p\left({\mathrm{AND}}_{H}\left(\rho_{\langle n,m\rangle }\right)\right)=p\left(\rho_{n}\right)\left(1-p\left(\rho_{n}\right)\right)$, which is the probability value of the syntactic contradiction given in Equation (24). This motivates the following notion of holistic contradiction.

**Definition** **6.** *An expression of the form ${\mathrm{AND}}_{Hol}\left(\rho_{\langle n,m\rangle }\right)$ is said to be an holistic contradiction whenever $\rho_{\langle m,k\rangle }\in D_{Hol}^{cont}$.*

**Example** **3.** *Let us consider the subfamily of three-parameter qubit states $\rho_{1bc}$ introduced in Example 2. Since ${\rho_{1bc}}_{\langle 1,1\rangle }\in D_{Hol}^{cont}$, then ${\mathrm{AND}}_{Hol}\left({\rho_{1bc}}_{\langle 1,1\rangle }\right)$ is an holistic contradiction. By the basic properties of $\rho_{1bc}$ mentioned in Example 1, we have that:*

*1.*  *
$p\left({\mathrm{AND}}_{H}\left({\rho_{1bc}}_{\langle 1,1\rangle }\right)\right)=\frac{1-1}{4}=0$
*
*2.*  *
$p\left(\mathrm{AND}(\rho_{abc_{1}}⊗\rho_{abc_{2}})\right)=p\left(\rho_{abc_{1}}\right)p\left(\rho_{abc_{2}}\right)=\frac{{\left(1-2\right)}^{2}-b^{2}}{16}=\frac{1-b^{2}}{16},$
*
*3.*  *$p\left(F^{1,1,1}\left(M\left(\rho_{abc}\right)⊗P_{0}\right)\right)=\frac{b^{2}-1}{16}$.*

**Figure 5:**
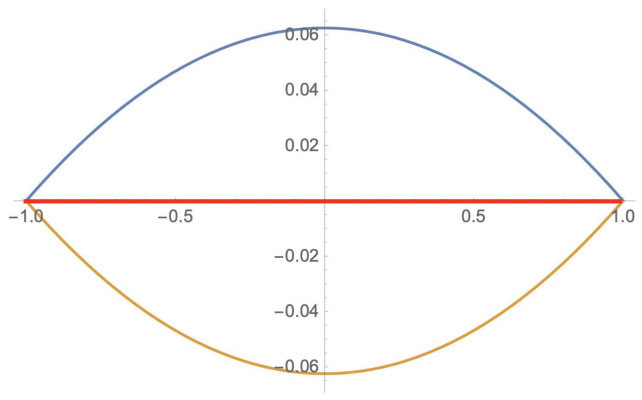
Holistic contradiction on ${\rho_{1bc}}_{\langle 1,1\rangle }$.

In Figure 5, the red line represents the value of the probability of the holistic contradiction over the family ${\rho_{1bc}}_{\langle 1,1\rangle }$ for each possible value of *b*, which is 0 in accordance with item 1 above. The blue line represents the probability contribution given by the partial traces of ${\rho_{1bc}}_{\langle 1,1\rangle }$ for each possible value of *b*; analogously, the brown line represents the (negative) contribution given by the holistic component of ${\rho_{1bc}}_{\langle 1,1\rangle }$ for each possible value of *b*.

## Data Availability

Not applicable.
